# Intake of the Total, Classes, and Subclasses of (Poly)phenols and Breast Cancer Risk: A Prospective Analysis of the EPIC Study

**DOI:** 10.3390/antiox15030342

**Published:** 2026-03-09

**Authors:** María Fernanda López-Padilla, David Seoane-Miraz, Daniel Guiñón-Fort, Enrique Almanza-Aguilera, Christina C. Dahm, Mariem Louati-Hajji, Claire Cadeau, Francesca Mancini, Rashmita Bajracharya, Verena Katzke, Matthias B. Schulze, Giovanna Masala, Claudia Agnoli, Simona Signoriello, Lisa Padroni, Cristina Lasheras, María-José Sánchez, Amaia Aizpurua Atxega, Sandra M. Colorado-Yohar, Alba Gasque, Wing Ching Chan, Yahya Mahamat Saleh, Anne Tjønneland, Christina M. Lill, Marta Farràs, Raul Zamora-Ros

**Affiliations:** 1 Unit of Nutrition and Cancer, Cancer Epidemiology Research Program, Catalan Institute of Oncology (ICO), Bellvitge Biomedical Research Institute (IDIBELL), 08908 L’Hospitalet de Llobregat, Barcelona, Spain; mflopez@idibell.cat (M.F.L.-P.); dseoanem@uoc.edu (D.S.-M.); dguinon@idibell.cat (D.G.-F.); ealmanzaa@outlook.com (E.A.-A.); 2 Departament de Nutrició, Ciències de l’Alimentació i Gastronomia, Facultat de Farmàcia i Ciències de l’Alimentació, Universitat de Barcelona (UB), 08028 Barcelona, Spain; 3 Department of Public Health, Aarhus University, DK-8000 Aarhus, Denmark; ccd@ph.au.dk; 4 Paris-Saclay University (UVSQ), Univ. Paris-Sud, 91190 Gir-sur-Yvette, France; mariem.hajji@inserm.fr (M.L.-H.); claire.cadeau@gustaveroussy.fr (C.C.); francesca.mancini@gustaveroussy.fr (F.M.); 5 Inserm, Gustave Roussy, Exposome and Heredity Team, Centre for Research Epidemiology and Population Health (CESP), 94800 Villejuif, France; 6 Department of Cancer Epidemiology, German Cancer Research Center (DKFZ), 69120 Heidelberg, Germany; rashmita.bajracharya@dkfz-heidelberg.de (R.B.); v.katzke@dkfz-heidelberg.de (V.K.); 7 Department of Molecular Epidemiology, German Institute of Human Nutrition Potsdam-Rehbruecke, 14558 Nuthetal, Germany; mschulze@dife.de; 8 Institute of Nutritional Science, University of Potsdam, 14558 Nuthetal, Germany; 9 Clinical Epidemiology Unit, Institute for Cancer Research, Prevention, and Clinical Network (ISPRO), 50139 Florence, Italy; g.masala@ispro.toscana.it; 10 Epidemiology and Prevention Unit, Fondazione IRCCS Istituto Nazionale dei Tumori di Milano, 20133 Milan, Italy; claudia.agnoli@istitutotumori.mi.it; 11 Medical Statistics Unit, University “L. Vanvitelli”, 81100 Naples, Italy; simona.signoriello@unicampania.it; 12 Department of Clinical and Biological Science, University of Turin, 10043 Orbrassano, Italy; lisa.padroni@unito.it; 13 Department of Functional Biology, University of Oviedo, 33007 Oviedo, Spain; lasheras@uniovi.es; 14 Escuela Andaluza de Salud Pública (EASP), 18011 Granada, Spain; mariajose.sanchez.easp@juntadeandalucia.es; 15 Instituto de Investigación Biosanitaria IBS. Granada, 18012 Granada, Spain; 16 Centro de Investigación Biomédica en Red de Epidemiología y Salud Pública (CIBERESP), 28029 Madrid, Spain; scyohar@gmail.com; 17 Biogipuzkoa Health Research Institute, Group of Epidemiology of Chronic and Communicable Diseases, 20014 San Sebastian, Gipuzkoa, Spain; a-aizpuruaatxega@euskadi.eus; 18 Ministry of Health of the Basque Government, Sub-Directorate for Public Health and Addictions of Gipuzkoa, 20013 San Sebastian, Gipuzkoa, Spain; 19 Department of Epidemiology, Murcia Regional Health Council, IMIB-Arrixaca, 30007 Murcia, Spain; 20 Research Group on Demography and Health, National Faculty of Public Health, University of Antioquia, Medellin 050001, Colombia; 21 Instituto de Salud Pública y Laboral de Navarra, 31003 Pamplona, Spain; alba.gasque.satrustegui@navarra.es; 22 Navarra Institute for Health Research (IdiSNA), 31008 Pamplona, Spain; 23 Nuffield Department of Population Health, University of Oxford, Oxford OX3 7BN, UK; stephanie.chan@ndph.ox.ac.uk; 24 Nutrition and Metabolism Branch, International Agency for Research on Cancer, 69007 Lyon, France; mahamaty@iarc.who.int; 25 Danish Cancer Institute, DK-2100 Copenhagen, Denmark; annet@cancer.dk; 26 Institute of Epidemiology and Social Medicine, University of Münster, 48149 Münster, Germany; c.lill@imperial.ac.uk; 27 Ageing and Epidemiology Unit (AGE), School of Public Health, Imperial College London, London SW7 2AZ, UK

**Keywords:** (poly)phenols, flavonoids, breast cancer, diet, cohort, EPIC study

## Abstract

Polyphenols represent the largest and most diverse class of dietary antioxidants. Epidemiological evidence linking specific (poly)phenol classes, such as flavonoids and lignans, to breast cancer (BC) risk remains limited and largely inconclusive in prospective studies. The aim of this study is to examine the association between the intake of total (poly)phenols—and its classes and subclasses—and BC risk—overall and by subtypes (estrogen, progesterone, and human epidermal growth factor receptor 2 (HER2))—in the European Prospective Investigation into Cancer and Nutrition (EPIC) cohort. The EPIC cohort includes 257,960 adult women from seven European countries. During a mean follow-up of 14 years, there were 10,722 incident overall BC cases. Associations were computed using Cox regression models adjusted for potential confounders. No significant associations were found between total (poly)phenol intake and overall BC risk (HR_Q5 vs. Q1_ = 1.02; 95% CI: 0.95–1.11). In addition, null associations were mostly found between classes and subclasses of (poly)phenols and BC subtypes. After stratifying by menopausal status, no significant associations were observed. In conclusion, this study found no evidence of associations between the intake of any class or subclass of (poly)phenols and BC risk in the European population.

## 1. Introduction

(Poly)phenols are secondary metabolites synthesized by plants to protect themselves against environmental stress. (Poly)phenols are a large and complex group of compounds, comprising around 500 molecules found in usual foods, divided into four main classes depending on their chemical structure: flavonoids, phenolic acids, lignans, and stilbenes. There are more than 25 subclasses [1]. In Europe, their total mean intake is approximately 1 g/day, making them the most abundant bioactive compounds in habitual human diets [2,3]. Experimental and observational evidence has shown several potential beneficial properties of (poly)phenols against chronic diseases, including breast cancer (BC) development [4]. (Poly)phenols are potent in vitro antioxidants due to their chemical structure, with hydroxyl groups acting as electron or hydrogen donors to neutralize free radicals and reactive oxygen species (ROS) [5]. However, their direct antioxidant capacity is questionable due to low blood concentrations, with their effects mainly linked to antioxidant enzyme activation [6]. In addition to their antioxidant and anti-inflammatory effects, (poly)phenols may exhibit specific anticancer properties by enhancing signaling pathways related to cell cycle arrest and apoptosis that inhibit cell proliferation and migration [7]. They may also protect against epigenetic dysregulation in breast cancer development by inhibiting DNA methyltransferase, altering chromatin modification, and regulating tumor suppressor gene expression [8]. Furthermore, (poly)phenols modulate the microbiota and prevent gut dysbiosis [9], which has been linked to BC development and prognosis [10].

BC is the most common cancer among women worldwide, with its incidence continuously rising, particularly in highly developed countries [11]. Molecular subtypes are classified based on progesterone, estrogen, and HER2 receptor status, which influence disease progression, treatment response, and survival outcomes [12]. Several modifiable and non-modifiable risk factors are known to increase BC risk, including unhealthy diet [13]. Notably, (poly)phenol-rich diets have been associated with a lower BC risk, especially in postmenopausal women [14]. However, epidemiological evidence linking specific (poly)phenol classes, such as flavonoids and lignans, to BC risk remains limited and largely inconclusive in prospective studies. To our knowledge, no prospective studies have yet explored associations with other subclasses, particularly phenolic acids and minor classes [14]. In previous research of our group, using data from the European Prospective Investigation into Cancer and Nutrition (EPIC) study, no statistically significant associations were observed between the intake of flavonoids and lignans and overall BC risk, nor by estrogen and progesterone receptor subtype [15]; but no data was available at that time on the HER2 receptor and on other classes and subclasses of (poly)phenols. Therefore, the aim of this study was to update the previous relationships with more BC cases, expand the research to more molecular BC subtypes (such as triple negative BC), and include all the known (poly)phenol classes and subclasses present in human diets.

## 2. Materials and Methods

### 2.1. Study Population

The EPIC study is an ongoing multicenter prospective cohort aimed at evaluating the associations between dietary, lifestyle, and genetic factors and cancer risk [16]. All participants were enrolled between 1992 and 2000 from 23 centers in 10 European countries. Overall, 367,903 female participants were recruited from the general population from defined areas in each country with the exception of women who were members of a health insurance program for state school employees (France), women attending breast cancer screening (Utrecht, the Netherlands and Florence, Italy), blood donors (some centers in Italy and Spain) and vegetarians (the ‘health conscious’ cohort in Oxford, UK), of whom 33,054 were excluded because of prevalent cancer at baseline, missing information on dietary or lifestyle variables, or due to an extreme ratio between energy intake and energy requirement. Data from Greece, Norway and Sweden was not available (n = 76,715) for this study. In total, 257,960 women from France, Italy, Spain, United Kingdom, The Netherlands, Germany and Denmark were included in the analyses. The ethical review boards of the International Agency for Research on Cancer (IARC) and all participating centers approved the study protocol, and all participants provided written informed consent.

### 2.2. Follow-Up and Case Assessment

Regional and national population-based cancer registries were used for BC incidence cases in Denmark, Italy, The Netherlands, Spain and The United Kingdom. Active follow-up of cases was carried out through health insurance records, cancer and pathology registries and direct contact with the next of kin for Germany, Naples, and France. Pathology reports were used for cancer diagnosis for all EPIC centers. Follow-up was from study entry until whichever occurred first: cancer diagnosis (except nonmelanoma skin cancer), death, emigration, or the end of follow-up, which varied across centers as reported in previous publications. The mean of follow-up was 14 years. Complete follow-up censoring dates varied among centers, ranging between June 2008 and December 2013 [15].

### 2.3. Dietary and Lifestyle Collection

At baseline, the usual diet during the previous 12 months was quantified using validated country/center-specific dietary questionnaires [17]. Energy and nutrient intakes were estimated using the EPIC nutrient database [18]. All classes, subclasses, and individual (poly)phenols have been calculated using the Phenol-Explorer database [19]. At baseline, a standardized lifestyle questionnaire was administered with information on sociodemographics, smoking, alcohol consumption, physical activity [20], education, and medical history. Additionally, anthropometric data were mostly measured in all centers at recruitment, except in Oxford (UK) and France where they were self-reported.

### 2.4. Statistical Analysis

(Poly)phenol intake was divided into quintiles, according to the intake distribution among all participants. For continuous distribution, (poly)phenol intakes were log-2 transformed to reduce skewness; each increase of one unit corresponds to doubling the intake. P-trend was performed by replacing the polyphenol value with their mean within each quintile, creating a variable with five values. This new variable was incorporated into Cox proportional hazards models to estimate the hazard ratio (HR) for each. The HR and 95% confidence interval (CI) from Cox regression models were used to investigate the associations between dietary (poly)phenol intake and overall BC risk and by clinical subtype with age as the underlying time variable in all models. Entry time was age at recruitment and exit time was age at diagnosis, death, or censoring date (lost or end of follow-up), whichever occurred first. The proportional hazards assumption was evaluated in all models by using the analysis of Schoenfeld residuals, and no evidence of violation was detected. A total of 3 models were constructed. Model 1 was stratified by center and age (per 1 y interval) at baseline. Model 2 was further adjusted for smoking status (never, former, current smoker, and unknown), educational level (none, primary school, technical/professional school, secondary school, university or higher, and not specified), alcohol consumption (no consumption, <5, 5–10, 10–20, 20–40, and >40 g/day), physical activity (inactive, moderately inactive, moderately active, active, and not specified), body mass index (BMI; kg/m^2^), menopausal status (pre-, peri- and postmenopausal), previous use of hormonal treatment (no, yes, or unknown), and previous use of oral contraceptives (no, yes, or unknown), total energy intake (kcal/d), total fiber intake (continuous, g/d), and height (cm). An interaction term between menopausal status and BMI (<25.0 and ≥25.0 kg/m^2^) was introduced to take into account the differential effect of excess body weight in BC risk before or after menopause. Model 3 was additionally adjusted for menopause status, and BMI and menopause interaction.

All covariates included in the models were based on a priori assumptions [15]. In addition, (poly)phenol intakes were included in the statistical models as energy density (mg/2000 kcal/d) and using the Willet’s residual model [21]. The results in all three strategies were almost identical, so only results with the traditional strategy adjusted for energy are presented.

Possible interactions between (poly)phenol intake and smoking status, BMI (<25.0 and ≥25.0 kg/m^2^), and menopausal status on BC risk were examined. The likelihood ratio test was used to evaluate these interactions. Separate models were defined to assess the risk of BC by clinically relevant sub-types (ER, PR, and HER2 status, and their combinations). The Wald test was used to evaluate the heterogeneity of the risk between clinical BC subtypes. Sensitivity analyses were conducted excluding 1218 total BC cases diagnosed during the first 2 years of the follow-up. All results with a *p*-value < 0.05 (two-sided test) were considered statistically significant. The Bonferroni correction method was used to adjust *p*-values for multiple comparisons. Then, results were considered statistically significant if *p* < 0.002 (*p* < 0.05/23, the number of tests for the intakes of all (poly)phenol classes and subclasses was (23)). Statistical analyses were carried out using R (version 4.2.1) and RStudio (version 2022.07.1) software.

## 3. Results

The study included a total population of 257,960 women, of whom 10,772 were diagnosed with BC during the mean of 14 y follow-up. Among the BC subtypes, ER+ and PR+ were the most common (62.9% and 41.14%), whereas triple-negative BC was the least frequent (2.7%). France had the highest number of BC cases (30.7%). Italy showed a high proportion of ER-positive cases (840 of 1211; 69.4%), whereas Denmark, despite having a larger number of breast cancer cases overall (1867), showed a lower proportion of ER-positive tumors (1167; 62.5%), reflecting distinct subtype distributions between countries (Table 1).

The Supplementary Tables S1 and S2 show the baseline characteristics of the study population by quintiles of (poly)phenol intake. BMI was slightly lower in the participants in the highest quintile of (poly)phenol intake compared to those in the first quintile. Women in the higher quintiles of (poly)phenol intakes tend to have a higher education level, be more physically active, consume more tobacco, have a higher fiber intake and have a higher proportion of both hormone replacement therapy (HRT) and contraceptive pill users than those in the lower quintiles.

Table 2 shows the associations between total (poly)phenol intake and BC risk using the multivariable Cox models progressively adjusting for potential confounding factors, such as lifestyle and dietary habits. No associations were observed with overall BC risk (HR_Q5 vs. Q1_ = 1.02; 95% CI: 0.95–1.10). Although a few borderline associations were detected in individual quintiles for certain receptor subtypes (ER-negative: HR = 1.20, 95% CI 0.98–1.46; triple-negative: HR = 1.66, 95% CI 1.06–2.61), overall, no consistent associations were found between total (poly)phenol intake and risk of BC subtypes by hormone receptor (PR, ER) and HER2 status. No interactions with either menopausal status, smoking status, or BMI were observed.

**Figure 1:**
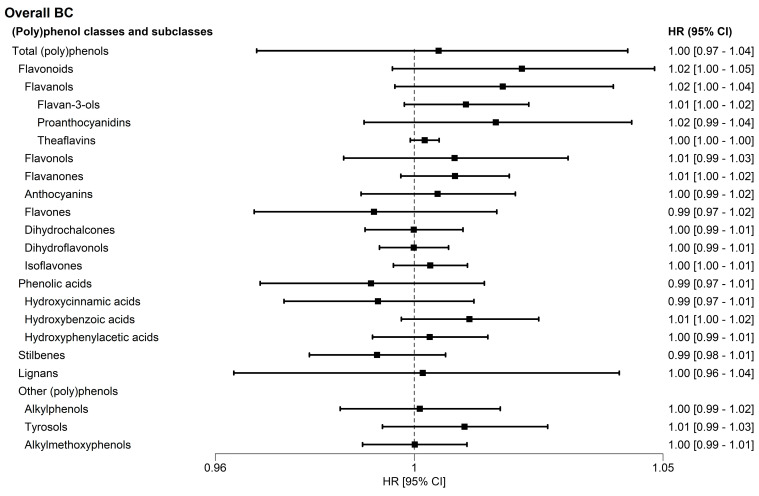
Adjusted Hazard Ratio for overall breast cancer, according to doubling all classes and subclasses of (poly)phenol intakes (log_2_ mg/day) in the European Prospective Investigation into Cancer and Nutrition Study. Model 3: stratified by age and center, and adjusted for education level, smoking intensity, physical activity, body mass index (BMI; kg/m^2^), alcohol intake (g/day), hormone replacement therapy (yes/no/unknown), use of oral contraceptives (yes/no/unknown), total energy intake (kcal/day), fiber intake (g/day), and height (cm).

**Figure 2:**
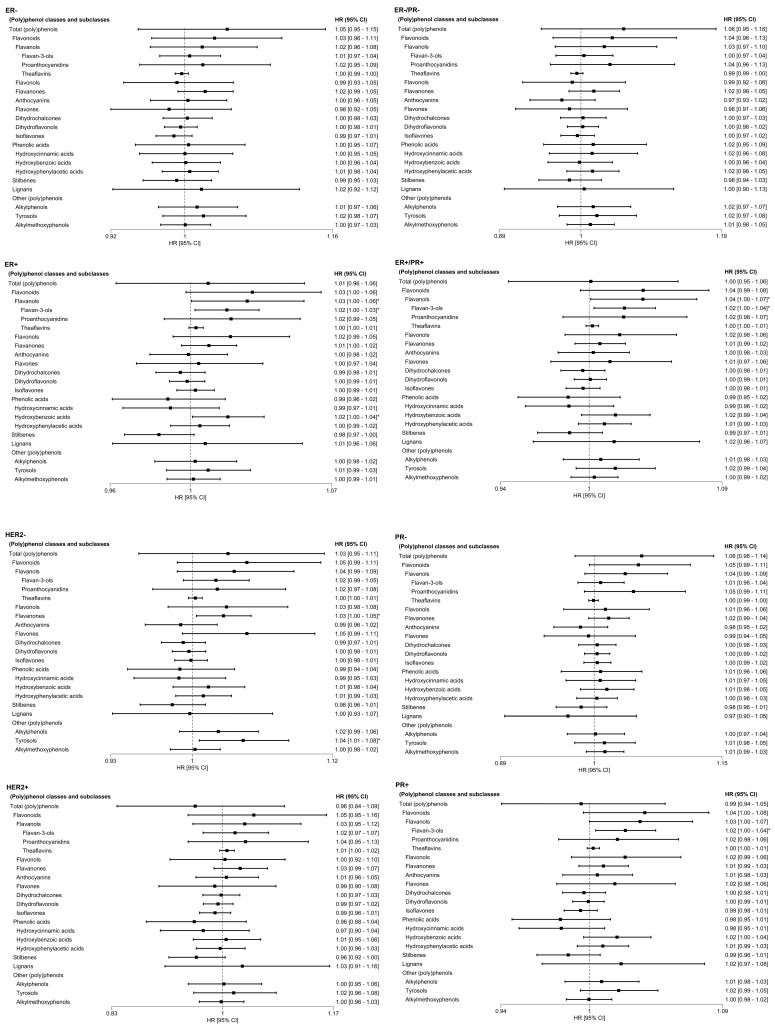

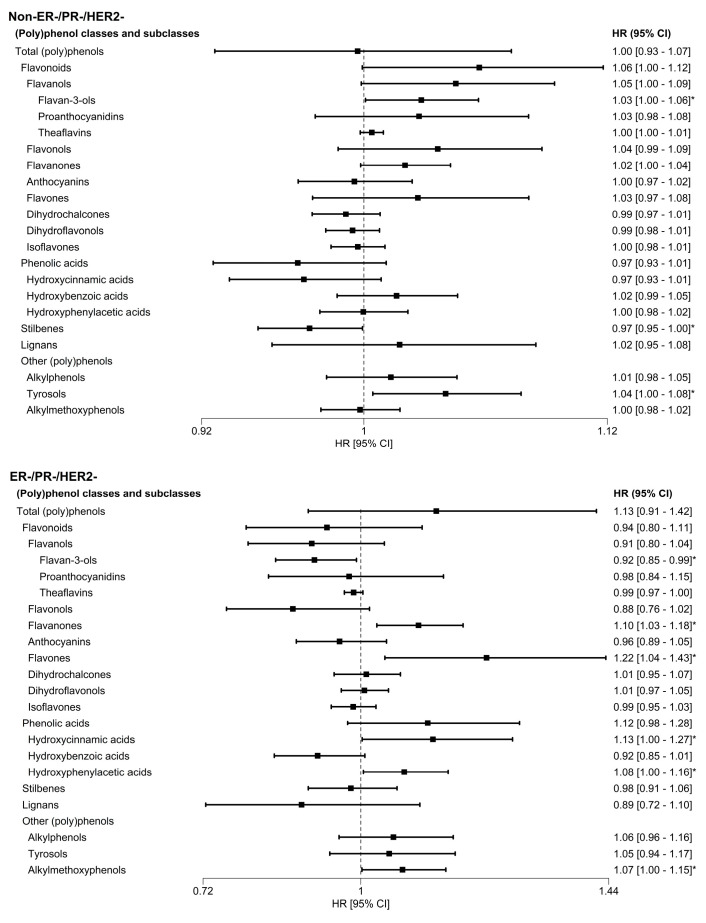
Adjusted HR for BC subtypes, after doubling all classes and subclasses of (poly)phenol intakes (log_2_ mg/day) in the European Prospective Investigation into Cancer and Nutrition Study. Model 3: stratified by age and center, and adjusted for education level, smoking intensity, physical activity, body mass index (BMI; kg/m^2^), alcohol intake (g/day), hormone replacement therapy (yes/no/unknown), use of oral contraceptives (yes/no/unknown), total energy intake (kcal/day), fiber intake (g/day), and height (cm). *p*-value < 0.05 (*).

Figure 1 shows the relationship between the doubling intake of all classes and subclasses of (poly)phenols and the risk of overall BC in the EPIC cohort. None of the associations were statistically significant. After stratifying by menopausal status, neither class nor subclass of (poly)phenol intakes were associated with overall BC risk in premenopausal women. In postmenopausal women, slight borderline trends were observed in flavonoids, flavonols, flavan-3-ols and hydroxybenzoic acids that tend to increase the overall BC risk, but these associations did not exceed the multiple comparison correction threshold (Bonferroni correction) (Figure S1). When stratified by BC subtypes, all associations with all classes and subclasses of (poly)phenol intakes were null. Borderline negative and positive associations between a number of polyphenol subclasses and BC risk were observed mainly in triple-negative BC subtype, but none of these results maintain the statistical significance after applying the Bonferroni correction (Figure 2).

## 4. Discussion

Epidemiological studies have suggested that dietary factors may play an important role in BC development [13], especially the protective effect of plant-based foods, probably due to their (poly)phenols [22] which are antioxidants. To our knowledge, this is the first time that the associations between total and classes and subclasses of (poly)phenols and the risk of overall BC and its subtypes were prospectively analyzed.

(Poly)phenols represent the largest and most diverse class of dietary antioxidants, and their antioxidant effect has been reported to be produced by phenolic acids by scavenging free radicals, inactivating enzymes related to ROS production, and activating antioxidant enzymes. The neutralization of free radicals is due to the hydroxyl groups in their structure. Polyphenols have been shown to regulate oxidative stress signaling pathways and downregulate the stress-activated MAPK pathway leading to the inhibition of ROS production, oxidative stress injury, and apoptosis-related pathways. In addition, (poly)phenols could induce apoptotic cell death in preneoplastic cells through various growth inhibitory mechanisms such as the activation of cytochrome c and caspases, the arrest of the cell cycle, and the modulation of signaling pathways (NF-κB, JAK/STAT) which result in the inhibition of tumor progression [23,24,25].

In our study, no associations were found between the intake of total (poly)phenol or any of its classes and subclasses and overall BC risk or any of its subtypes. These findings are consistent with the results reported by Liu et al., who also did not observe an association between polyphenol intake and BC risk [26]. In addition, in a meta-analysis performed by Grosso et al., no significant associations with total flavonoids were observed [27]. Indeed, in a previous analysis in the EPIC cohort, similar null associations were observed with either total flavonoids or its subclasses and BC risk [15] and prognosis [28]. These findings further support the lack of significant relationships between (poly)phenol intake and BC outcomes across different analyses. In addition, a study performed by Gardeazabal et al. showed no significant association between total (poly)phenol intake and overall BC risk in the Spanish SUN cohort [22].

Although the association of poly(phenols) with overall breast cancer and its subtypes were largely statistically not significant in prospective cohort studies, in a smaller Spanish case–control study (23), the intake of stilbenes, hydroxybenzaldehydes, and hydroxycoumarins were associated with a lower BC risk in all women, independently of the menopausal or hormonal receptor status [29]. In line with this, a study suggested a reduced BC risk of 19% with high green tea consumption compared with lower green tea consumption [30]; however, non-significant differences were observed in other cohort studies [31]. In contrast, results from a meta-analysis which combined cohort and case–control studies suggested that total flavonoids, flavonols, flavones, flavan-3-ols, flavanones, and isoflavones were associated with a lower overall BC risk [27]. These last results are comparable to our results with flavanols, since tea is the main source of flavanols in Europe [2,32].

In our study, when stratified by menopausal status, null results were observed among postmenopausal and premenopausal women as in our previous EPIC analysis on flavonoids and lignans [15]. In contrast, in a previous case–control study, inverse associations regarding lignan intake and BC risk were reported in all menopausal statuses [29], whereas in the meta-analysis performed by Grosso G et al., only proanthocyanidin intake was associated with a lower BC risk in postmenopausal women, but not in premenopausal women [27]. Similarly, in the study performed by Gardeazabal I et al., inverse associations were observed with flavonoids, flavonols, flavan-3-ols and hydroxybenzoic acids in postmenopausal women, but not in premenopausal women [22].

In our study, direct association trends were observed for some tumor subtypes, with the strongest trend observed in the triple-negative BC subtype. Similar patterns were seen when stratified by menopausal status for postmenopausal women, although they were not significant after applying the Bonferroni correction. Further research is needed to clarify this potential impact of (poly)phenols in postmenopausal women and in the triple-negative subtype [2].

Despite numerous studies and meta-analyses, the results regarding the impact of (poly)phenols on BC risk remain uncertain. For instance, while a number of studies have observed a reduced BC risk associated with high intake of specific (poly)phenols like flavonols and flavones [27,33], other studies have found no significant associations between BC risk and these same (poly)phenol classes [15,22], highlighting inconsistencies in the evidence. Furthermore, some protective associations appear to vary by menopausal status and tumor receptor subtypes, as observed in a similar study based in the Southern Community in the United States, a population that is 66.5% African American. For total (poly)phenol intake they observed a reduced risk of BC incidence in ER/PR-positive subtype and between phenolic acids and overall BC in postmenopausal women [34]. These findings add complexity to the overall understanding and suggest potential differences by ethnicity [34], particularly considering that predominantly Caucasian participants were recruited to EPIC. Notably, in our analysis, we found no significant associations between any (poly)phenol intake and overall BC risk, emphasizing the need for further research to clarify these findings and determine the specific conditions (type of cancer, menopausal status) under which (poly)phenols might confer protective effects against BC. This underscores the importance of continued investigation into dietary factors and their role in cancer prevention, considering individual variability and the complex interplay of various biological mechanisms.

Our study has both strengths and limitations. Among its strengths are the prospective design, the large sample size and number of cases, the long follow-up period, and the inclusion of several European countries with a large dietary heterogeneity. Another strength of this study is the availability of data related to all relevant clinical BC subtypes as well as dietary data of all (poly)phenol subclasses. However, there are also some limitations. Potential measurement errors in dietary intake assessments could weaken any true association between (poly)phenol intake and BC risk. Specifically, self-reported dietary questionnaires may introduce bias into the (poly)phenol intake assessment due to random and systematic measurement errors, despite being validated in each center/country [16]. Another limitation of this study is that we only have baseline data, which does not allow us to assess changes over the follow-up period. Additionally, there is a potential risk of confounding bias, since several lifestyle and other dietary factors related to BC were different according to polyphenol intake. Although we have included them in the statistical models, measurement error and changes during follow-up may affect our results. Furthermore, while Phenol-Explorer is the most comprehensive database available, it has missing data for certain foods and polyphenols, which could impact the accuracy of intake estimations. Another limitation is the potential modification of diet during the early pre-diagnostic period of the disease; however, sensitivity analyses excluding incident cases diagnosed in the first 2 years of follow-up did not alter the associations. In addition, it is necessary to consider that multiple comparisons were performed in this study, and when Bonferroni multiple comparisons test was applied, the significant results lost their statistical significance. The findings of this study cannot be generalized to other racial/ethnic groups, as the study population consisted almost exclusively of Caucasian participants.

## 5. Conclusions

This study did not find associations between total (poly)phenol intake, including its classes and subclasses, and BC risk or its molecular subtypes. While (poly)phenols are recognized for their potential anti-inflammatory, antioxidant, and anticancer properties, our findings are aligned with previous cohort studies reporting no significant associations. Comprehensive analyses and sensitivity tests consistently showed null results across hormone receptor and HER2 subtypes. These findings highlight the need for further research into dietary factors, individual variability, and tumor biology in BC prevention.

## Figures and Tables

**Table 1:** Distribution of participants and breast cancer cases by countries in the EPIC study.

Country	n	BC	ER+	ER-	PR+	PR-	HER2+	HER2-	ER+/PR+	ER-/PR-	ER+/PR+/HER+	ER-/PR-/HER2-
France	67,300	3308	2324 (34.2%)	565 (36.9%)	1766 (39.5%)	1025 (43.6%)	184 (22.4%)	754 (31%)	1655 (38.9%)	446 (39.1%)	63 (15.1%)	92 (31.6%)
Italy	30,498	1211	840 (12.4%)	182 (11.9%)	681 (15.2%)	325 (13.8%)	173 (21%)	482 (19.8%)	640 (15%)	141 (12.4%)	97 (23.3%)	52 (17.9%)
Spain	24,842	655	369 (5.4%)	90 (5.8%)	314 (7.0%)	133 (5.6%)	130 (15.8%)	202 (8.3%)	298 (7%)	72 (6.3%)	86 (20.7%)	27 (9.3%)
United Kingdom	52,489	1874	973 (14.3%)	189 (12.3%)	289 (6.4%)	165 (7.0%)	98 (11.9%)	443 (18.2%)	286 (6.7%)	107 (9.4%)	26 (6.3%)	51 (17.5%)
The Netherlands	26,828	1046	560 (8.2%)	98 (6.4%)	431 (9.6%)	218 (9.2%)	165 (20.1%)	232 (9.5%)	423 (9.9%)	92 (8.1%)	110 (26.4%)	30 (10.3%)
Germany	27,312	811	547 (8.0%)	138 (9.0%)	497 (11.1%)	187 (7.9%)	70 (8.5%)	317 (13%)	477 (11.2%)	118 (10.3%)	34 (8.2%)	39 (13.4%)
Denmark	28,691	1867	1167 (17.2%)	267 (17.4%)	486 (10.8%)	297 (12.6%)	-	-	472 (11.1%)	165 (14.5%)	-	-
All	257,960	10,772	6780 (62.9%)	1529 (14.2%)	4464 (41.4%)	2350 (21.8%)	820 (7.6%)	2430 (22.6%)	4251 (39.5%)	1141 (10.6%)	416 (3.9%)	291 (2.7%)

Abbreviations: BC, Breast Cancer; ER+, Estrogen Receptor Positive; ER-, Estrogen Receptor Negative; PR+, Progesterone Receptor Positive; PR-, Progesterone Negative; HER2+, Human Epidermal Growth Factor 2 Positive; HER2-, Human Epidermal Growth Factor 2 Negative.

**Table 2:** Adjusted Hazard Ratio for overall and breast cancer (BC) subtypes, according to quintiles of total (poly)phenol intakes (mg/day) in the European Prospective Investigation into Cancer and Nutrition Study.

Total (Poly)phenols	n Cases	Quintile 1	Quintile 2	Quintile 3	Quintile 4	Quintile 5	P-Trend
		HR (95% CI)	HR (95% CI)	HR (95% CI)	HR (95% CI)	HR (95% CI)	
Cut-offs of intake (mg/d)		778	1.053	1.326	1.676	10.615	
N of participants	257,960	51,592	51,592	51,592	51,592	51,592	
Basic model ^1^	10,772	Referent	1.00 (0.93, 1.07)	1.07 (1.00, 1.15)	1.07 (1.00, 1.14)	1.09 (1.01, 1.17) *	0.01 *
Multivariable model ^3^	10,772	Referent	0.97 (0.91, 1.04)	1.03 (0.96, 1.11)	1.02 (0.95, 1.09)	1.02 (0.95, 1.10)	0.32
Menopausal status ^2^							
Premenopausal BC	2831	Referent	1.06 (0.94, 1.19)	1.04 (0.92, 1.19)	1.09 (0.95, 1.25)	1.03 (0.89, 1.19)	0.89
Postmenopausal BC	5827	Referent	0.92 (0.84, 1.02)	1.02 (0.92, 1.12)	1.01 (0.91, 1.11)	1.00 (0.90, 1.11)	0.47
*p*-value for interaction ^4^							0.14
BC by Hormone receptors status ^3^							
ER(+)	6780	Referent	0.95 (0.87, 1.03)	1.01 (0.93, 1.10)	1.02 (0.93, 1.12)	1.02 (0.93, 1.12)	0.27
ER(−)	1529	Referent	1.08 (0.90, 1.30)	1.23 (1.03, 1.48) *	1.00 (0.82, 1.22)	1.20 (0.98, 1.46)	0.20
P-Wald test ^5^							0.45
PR(+)	4464	Referent	0.96 (0.87, 1.06)	1.05 (0.94, 1.16)	1.03 (0.92, 1.15)	0.99 (0.88, 1.11)	0.88
PR(−)	2350	Referent	1.01 (0.88, 1.17)	1.10 (0.95, 1.27)	1.08 (0.92, 1.25)	1.17 (1.00, 1.37)	0.04 *
P-Wald test ^5^							0.23
ER(+) PR(+)	4251	Referent	0.96 (0.87, 1.06)	1.04 (0.94, 1.15)	1.05 (0.94, 1.17)	0.99 (0.88, 1.12)	0.73
ER(−) PR(−)	1141	Referent	1.09 (0.88, 1.33)	1.22 (0.99, 1.50)	1.05 (0.84, 1.32)	1.23 (0.97, 1.54)	0.15
P-Wald test ^5^							0.41
HER2(+)	820	Referent	0.99 (0.80, 1.22)	1.10 (0.88, 1.38)	0.97 (0.75, 1.25)	0.82 (0.61, 1.11)	0.25
HER2(−)	2430	Referent	0.99 (0.87, 1.12)	0.98 (0.85, 1.12)	1.03 (0.89, 1.19)	1.14 (0.97, 1.33)	0.07
P-Wald test ^5^							0.36
Triple negative	291	Referent	1.26 (0.87, 1.83)	1.17 (0.78, 1.76)	1.02 (0.65, 1.61)	1.66 (1.06, 2.61) *	0.07
Non-triple negative	2666	Referent	0.97 (0.86, 1.09)	1.01 (0.89,1.14)	1.03 (0.89,1.18)	1.03 (0.88,1.20)	0.57
P-Wald test ^5^							0.39

^1^ basic model: stratified by age (1y) and center. ^2^ multivariable model 2: basic model adjusted for school level, smoke intensity, physical activity, BMI (kg/m^2^), alcohol intake (g/day), hormone replacement therapy (yes/no/unknown), oral contraceptives (yes/no/unknown), total energy intake (kcal/day), fiber intake (g/day), height (cm). ^3^ multivariable model 3: multivariable model 2 additionally adjusted for menopause status, and BMI and menopause interaction. ^4^ *p*-value for interaction is based upon the likelihood ratio (LR) test. ^5^ *p*-value for the Wald test assessing the homogeneity of the relative risks. *p*-value < 0.05 (*).

## Data Availability

For information on how to submit an application for gaining access to EPIC data and/or biospecimens, please follow the instructions at https://epic.iarc.fr/access/ (accessed on 15 January 2023).

## Supplementary Materials

The following supporting information can be downloaded at: https://www.mdpi.com/article/10.3390/antiox15030342/s1, Table S1: Baseline characteristics according to quintiles of total polyphenols intake in the EPIC study; Table S2: Sociodemographic and Lifestyle Characteristics according to quintiles of total polyphenol intake in the EPIC study; Figure S1: Adjusted Hazard Ratio for overall breast cancer, after doubling all classes and subclasses of (poly)phenol intakes (log_2_ mg/day) by menopause status in the European Prospective Investigation into Cancer and Nutrition Study. Model 2: stratified by age and center, and adjusted for education level, smoking intensity, physical activity, body mass index (BMI; kg/m^2^), alcohol intake (g/day), hormone replacement therapy (yes/no/unknown), use of oral contraceptives (yes/no/unknown), total energy intake (kcal/day), fiber intake (g/day), and height (cm). Significant *p*-value < 0.05 (*); Bonferroni-corrected *p*-value < 0.002 (**).

## Abbreviations

The following abbreviations are used in this manuscript:

BC	breast cancer
CI	confidence interval
EPIC	European prospective investigation into cancer and nutrition
ER	estrogen receptor
HER2	human epidermal growth factor 2
HR	hazard ratio
HRT	hormone replacement therapy
IARC	international agency for research on cancer
PR	progesterone receptor
ROS	reactive oxygen species
