# Supplementary material for: Intake of the Total, Classes, and Subclasses of (Poly)phenols and Breast Cancer Risk: A Prospective Analysis of the EPIC Study
Source: Antioxidants (Basel). 2026 Mar 9;15(3):342. doi: 10.3390/antiox15030342 (PMC13024573; doi:10.3390/antiox15030342)
Supplement: Supplementary file 1 [file antioxidants-15-00342-s001.zip › Table S1. Baseline characteristics according to quintiles of total polyphenols intake in the EPIC study..pdf]

Table S1. Baseline characteristics according to quintiles of total polyphenols intake in the EPIC study.

Baseline Characteristics	All n = 257,960		Q1 [< 778] n = 51,592		Q2 [779, 1050] n = 51,592		Q3 [1051, 1330] n = 51,592		Q4 [1331, 1680] n = 51,592		Q5 [>1680] n = 51,592	
	n	%	n	%	n	%	n	%	n	%	n	%
BMI												
<25 (kg/m2)	153,282	59.42	25,922	50.24	30,051	58.25	31,541	61.14	32,536	63.06	33,232	64.41
>=25 (kg/m2)	104,678	40.58	25,670	49.76	21,541	41.75	20,051	38.86	19,056	36.94	18,360	35.59
Education level												
None	10,688	4.14	7,227	14.01	2,030	3.93	817	1.58	379	0.73	235	0.46
Primary school completed	57,304	22.21	15,981	30.98	13,495	26.16	10,709	20.76	9,086	17.61	8,033	15.57
Technical/professional school	52,108	20.20	7,074	13.71	10,281	19.93	11,566	22.42	11,937	23.14	11,250	21.81
Secondary school	62,175	24.10	10,877	21.08	12,404	24.04	13,029	25.25	12,642	24.50	13,223	25.63
University degree	62,775	24.34	9,119	17.68	11,652	22.58	12,925	25.05	13,790	26.73	15,289	29.63
Unknown	12,910	5.00	1,314	2.55	1,730	3.35	2,546	4.93	3,758	7.28	3,562	6.90
Physical activity												
Inactive	58,084	22.52	18,045	34.98	12,245	23.73	9,777	18.95	9,291	18.01	8,726	16.91
Moderately inactive	92,420	35.83	18,526	35.91	19,072	36.97	18,445	35.75	18,447	35.76	17,930	34.75
Moderately active	62,080	24.07	9,454	18.32	11,776	22.83	13,057	25.31	13,452	26.07	14,341	27.80
Active	41,642	16.14	5,079	9.84	7,649	14.83	9,311	18.05	9,494	18.40	10,109	19.59
Unknown	3,734	1.45	488	0.95	850	1.65	1,002	1.94	908	1.76	486	0.94
Smoking status												
Never	149,292	57.87	34,180	66.25	30,423	58.97	29,134	56.47	28,679	55.59	26,876	52.09
Former	58,359	22.62	8,310	16.11	11,228	21.76	12,355	23.95	12,966	25.13	13,500	26.17
Smoker	45,313	17.57	8,548	16.57	9,168	17.77	9,109	17.66	8,730	16.92	9,758	18.91
Unknown	4,996	1.94	554	1.07	773	1.50	994	1.93	1,217	2.36	1,458	2.83
Alcohol												
non-consumers	38,233	14.82	15,793	30.61	8,085	15.67	5,844	11.33	4,497	8.72	4,014	7.78

< 5 g/d	93,784	36.36	20,577	39.88	19,697	38.18	18,795	36.43	18,221	35.32	16,494	31.97
5 to 10 g/d	43,272	16.77	6,006	11.64	8,139	15.78	9,300	18.03	9,865	19.12	9,962	19.31
10 to 20 g/d	45,544	17.66	5,862	11.36	9,000	17.44	9,550	18.51	10,386	20.13	10,746	20.83
20 to 40 g/d	28,699	11.13	2,851	5.53	5,593	10.84	6,460	12.52	6,572	12.74	7,223	14.00
>= 40 g/d	8,428	3.27	503	0.97	1,078	2.09	1,643	3.18	2,051	3.98	3,153	6.11
Hormone Replacement Therapy												
No	211,183	81.87	44,968	87.16	42,912	83.18	41,933	81.28	41,122	79.71	40,248	78.01
Yes	42,751	16.57	5,870	11.38	7,982	15.47	8,854	17.16	9,532	18.48	10,513	20.38
Unknown	4,026	1.56	754	1.46	698	1.35	805	1.56	938	1.82	831	1.61
Oral Contraceptive												
No	235,915	91.45	47,378	91.83	46,685	90.49	46,925	90.95	47,358	91.79	47,569	92.20
Yes	15,684	6.08	3,426	6.64	3,894	7.55	3,527	6.84	2,786	5.40	2,051	3.98
Unknown	6,361	2.47	788	1.53	1,013	1.96	1,140	2.21	1,448	2.81	1,972	3.82
Menopause Status												
Premenopausal	92,464	35.84	22,007	42.66	19,929	38.63	17,669	34.25	16,817	32.60	16,042	31.09
Postmenopausal	121,623	47.15	22,207	43.04	23,185	44.94	25,037	48.53	25,805	50.02	25,389	49.21
Perimenopausal	43,873	17.01	7,378	14.30	8,478	16.43	8,886	17.22	8,970	17.39	10,161	19.69

BMI is expressed in kg/m². Alcohol intake is measured in grams per day (g/d), total energy intake in kilocalories per day (kcal/day), fiber intake in grams per day (g/day). Hormone replacement therapy (HRT) and oral contraceptive use are categorized as yes/no/unknown.
