# Supplementary material for: Intake of the Total, Classes, and Subclasses of (Poly)phenols and Breast Cancer Risk: A Prospective Analysis of the EPIC Study
Source: Antioxidants (Basel). 2026 Mar 9;15(3):342. doi: 10.3390/antiox15030342 (PMC13024573; doi:10.3390/antiox15030342)
Supplement: Supplementary file 1 [file antioxidants-15-00342-s001.zip › Table S2. Sociodemographic and Lifestyle Characteristics according to quintiles of total polyphenols intake in the EPIC study..CORRECT.pdf]

Table S2. Sociodemographic and Lifestyle Characteristics according to quintiles of total polyphenols intake in the EPIC study.

Sociodemographic and Lifestyle Characteristics	All n = 257,960			Q1 [< 778] n = 51,592			Q2 [779, 1050] n = 51,592			Q3 [1051, 1330] n = 51,592			Q4 [1331, 1680] n = 51,592			Q5 [>1680] n = 51,592		
	n	Mean	SD	N	Mean	SD	n	Mean	SD	n	Mean	SD	n	Mean	SD	n	Mean	SD
BMI	257,960	24.85	4.39	51,592	25.79	4.86	51,592	24.93	4.41	51,592	24.68	4.26	51,592	24.47	4.11	51,592	24.38	4.11
Age at recruitment	257,960	50.91	10.00	51,592	49.54	9.95	51,592	50.34	10.00	51,592	51.28	10.10	51,592	51.68	10.24	51,592	51.69	9.50
Fiber	257,960	22.80	7.51	51,592	19.40	6.14	51,592	21.30	6.47	51,592	22.50	6.65	51,592	23.95	7.12	51,592	26.83	8.71
Energy	257,960	1,988.21	546.57	51,592	1,763.89	490.73	51,592	1,907.82	504.17	51,592	1,977.53	516.31	51,592	2,053.57	524.66	51,592	2,238.22	577.22
Alcohol	257,960	9.18	12.56	51,592	4.99	8.52	51,592	8.20	10.85	51,592	9.63	12.10	51,592	10.56	13.08	51,592	12.50	15.80
Vitamin C	257,960	132.41	64.17	51,592	112.55	55.05	51,592	123.82	58.19	51,592	130.85	58.85	51,592	138.87	61.63	51,592	155.96	76.44
Height	257.960	161.9	6.5	51,592	159.43	6.68	51,592	161.42	6.55	51,592	162.45	6.36	51,592	162.86	6.24	51,592	163.2	6.08

BMI is expressed in kg/m². Alcohol intake is measured in grams per day (g/d), total energy intake in kilocalories per day (kcal/day), fiber intake in grams per day (g/day). Height is expressed in cm.
